# An Aspartic Protease of the Scabies Mite *Sarcoptes scabiei* Is Involved in the Digestion of Host Skin and Blood Macromolecules

**DOI:** 10.1371/journal.pntd.0002525

**Published:** 2013-11-07

**Authors:** Wajahat Mahmood, Linda T. Viberg, Katja Fischer, Shelley F. Walton, Deborah C. Holt

**Affiliations:** 1 Global and Tropical Health Division, Menzies School of Health Research, Charles Darwin University, Darwin, Australia; 2 Infectious Diseases Program, Biology Department, QIMR Berghofer Medical Research, Herston, Australia; 3 School of Health and Sports Science, University of the Sunshine Coast, Sippy Downs, Australia; National Institute of Allergy and Infectious Diseases, United States of America

## Abstract

**Background:**

Scabies is a disease of worldwide significance, causing considerable morbidity in both humans and other animals. The scabies mite *Sarcoptes scabiei* burrows into the skin of its host, obtaining nutrition from host skin and blood. Aspartic proteases mediate a range of diverse and essential physiological functions such as tissue invasion and migration, digestion, moulting and reproduction in a number of parasitic organisms. We investigated whether aspartic proteases may play role in scabies mite digestive processes.

**Methodology/Principle Findings:**

We demonstrated the presence of aspartic protease activity in whole scabies mite extract. We then identified a scabies mite aspartic protease gene sequence and produced recombinant active enzyme. The recombinant scabies mite aspartic protease was capable of digesting human haemoglobin, serum albumin, fibrinogen and fibronectin, but not collagen III or laminin. This is consistent with the location of the scabies mites in the upper epidermis of human skin.

**Conclusions/Significance:**

The development of novel therapeutics for scabies is of increasing importance given the evidence of emerging resistance to current treatments. We have shown that a scabies mite aspartic protease plays a role in the digestion of host skin and serum molecules, raising the possibility that interference with the function of the enzyme may impact on mite survival.

## Author Summary

Scabies is an infectious disease of the skin caused by infestation with the parasitic mite *Sarcoptes scabiei*. It is a disease that has a considerable impact on humans and other animals, including livestock, wildlife and companion animals. Scabies mites burrow into the skin of their host, consuming host skin and blood molecules. Aspartic proteases play a key role in invasion and digestion processes in many parasitic organisms. We have identified a scabies mite aspartic protease and have shown that it is capable of digesting human haemoglobin, serum albumin, fibrinogen and fibronectin *in vitro*, indicating that it plays a role in mite digestive processes. This raises the possibility that interfering with the function of this digestive enzyme may impact on mite survival.

## Introduction

Scabies is a disease of worldwide significance, caused by infestation of the skin with the “itch mite” *Sarcoptes scabiei*. At the end of the 20^th^ century, the global burden of scabies in humans was estimated to be around 300 million cases worldwide [1]. Scabies is also a significant veterinary disease with *S. scabiei* a common parasite of economically important livestock, wildlife and companion animals [2]. Evidence of emerging scabies mite resistance to ivermectin [3], [4], [5] and permethrin [6], [7] emphasises the need to develop new anti-parasite therapies. This requires further understanding of scabies mite biology and host interaction.

Proteases play critical roles in the lifecycles of many parasites. As a result they are considered to be potential targets for the development of novel immunotherapeutic, chemotherapeutic, and serodiagnostic agents. A study investigating the presence of proteolytic activity in scabies mite extract detected phosphatase, esterase, aminopeptidase and glycosidase activity [8]. However no trypsin or chymotrypsin like serine protease activity was detected in the scabies mite extract, although an active serine protease sequence had previously been identified among scabies mite expressed sequence tags [9]. A recombinant active scabies mite serine protease, designated *Sar s* 3, was shown to have trypsin like activity. Recombinant *Sar s* 3 cleaves human filaggrin *in vitro* and co-localises to the mite gut with filaggrin, a key component of the stratum corneum [10]. The presence of cysteine protease activity in scabies mite extract has not been investigated however multiple active as well as putatively inactive cysteine proteases have been identified among scabies mite expressed sequence tags [11] and functional analysis of these molecules is underway [12].

Aspartic proteases, which have a pair of aspartic acid residues at their active site, are known to mediate a range of diverse and essential physiological functions such as tissue invasion and migration, digestion, moulting and reproduction in a number of parasitic organisms. The largest and most widely studied group of aspartic proteases is the A1 family, which includes the sub-families of pepsinogen, renin, cathepsin E and cathepsin D. Cathepsin D-like aspartic proteases are utilised as digestive enzymes by many haematophagous and phytophagous hemipterans, dipterans, and coleopterans [13]. Aspartic proteases play a key role in haemoglobin proteolysis in a number of parasites including ticks [14], *Plasmodium* [15], *Schistosoma* [16], *Onchocerca* [17], *Haemonchus* [18] and hookworms [19]. A cathepsin D like aspartic protease of the hookworms *Ancylostoma caninum* and *Necator americanus* is expressed in the oesophagus and intestine of the parasites and digests host haemoglobin [19]. *Schistosoma japonicum* and *Schistosoma mansoni* possess a cathepsin D like aspartic protease expressed predominantly in the gastroepidermis that digests haemoglobin in ingested blood at a pH range of 2.5–4.6 [16]. These cathepsin D molecules were also found to degrade host IgG, complement C3 and serum albumin at acidic pH, contributing to the evasion of the host immune response as it penetrates its host [20]. In the more closely related context of ectoparasites, extracts of the sheep scab mite *Psoroptes ovis* have been shown to have aspartic protease activity that is partially responsible for the digestion of host fibrinogen and fibronectin. The enzyme is thought to play an anti-coagulant role by degrading fibrinogen thus ensuring a continuous flow of serous exudate from host skin lesions for mite nutrition [21]. An aspartic protease from the cattle tick *Rhipicephalus microplus* was demonstrated to have pepstatin-sensitive activity against haemoglobin and it's involvement in vitellin degradation was the first characterisation of an aspartic protease involved in yolk degradation in an arthropod [22]. Another aspartic protease in the same organism, designated tick heme-binding aspartic proteinase, was later shown not only to degrade vitellin but also to possibly regulate its degradation by binding heme molecules on the surface of the enzyme [23]. An aspartic protease from the tick *Haemophysalis longicornis* expressed as a recombinant protein was able to digest haemoglobin and showed elevated expression in the salivary gland and the midgut in response to a blood meal [24].

Scabies mite extract has been shown to degrade fibronectin, fibrinogen and haemoglobin [8] however the enzymes responsible for this were not identified. The presence of aspartic protease activity in the scabies mite extract was not investigated and the role that scabies mite aspartic proteases may play in the degradation of host proteins has not been determined. This study aimed to investigate aspartic protease activity in the scabies mite and to determine the role aspartic proteases may play in the digestion of physiologically relevant host macromolecules.

## Methods

### Ethics statement

Human scabies mites were harvested from skin collected from crusted scabies patients admitted to the Royal Darwin Hospital, Northern Territory, Australia. All participants were given project information and provided written informed consent prior to skin collection. Human blood was collected with informed consent from a healthy volunteer. The project was approved by the Human Research Ethics Committee of the Northern Territory Department of Health and the Menzies School of Health Research, and was conducted in accordance with the Australian National Health and Medical Research Council's National Statement on Ethical Conduct in Human Research.

### Preparation of whole scabies mite extract

Human scabies mites were collected in pools of approximately 150 and stored in PBS at −80°C until protein extraction. The mites were then washed twice with PBS with centrifugation at 13000 g for 5 minutes, resuspended in 100 ul PBS and homogenised with a motorised pestle. The suspension was sonicated for 1 minute and the debris pelleted by centrifugation at 17400 g for 30 minutes at 4°C. The supernatant was recovered and the protein concentration determined by Bradford assay [25] (Bio-Rad Laboratories, Reagents Park, NSW, Australia).

### Aspartic protease activity assays using a fluorogenic substrate

The fluorogenic substrate MoCAc-Gly-Lys-Pro-Ile-Leu-Phe-Phe-Arg-Leu-Lys(Dnp)-D-Arg-NH (Sigma Aldrich, Castle Hill, NSW, Australia) was used to detect aspartic protease activity in the scabies mite extract. Each 200 ul reaction contained 100 mM sodium citrate pH 3.5, 20 µM fluorogenic substrate dissolved in DMSO, 19 µg scabies extract protein or 27.5 nM human cathepsin D (Sigma Aldrich) and 5 µM pepstatin A (Sigma Aldrich), where used. The reactions were incubated at 37°C for 5 minutes prior to the addition of the substrate, and for a further 5 minutes if pepstatin A was also used. The fluorescence units were recorded at 60 second intervals commencing after 10 minutes, for three hours at 37°C on a Victor3 fluorometer (Perkin Elmer, Glen Waverley, Vic, Australia) using 340 nm absorption and 405 nm emission filters.

### Aspartic protease activity assays using haemoglobin as the substrate

Haemoglobin was extracted from human blood by washing 20 mL of blood twice with three volumes of normal saline with red cells pelleted by centrifugation at 600 g for 10 minutes. Pelleted red cells were then lysed by resuspending in four volumes of hypotonic medium (0.1× PBS) and gently mixing for 10 minutes. Cell debris was removed by centrifugation at 14000 g for 30 minutes. Haemoglobin concentration was measured by Bradford assay and the purity assessed by running on a 15% SDS-PAGE gel and staining with coomassie brilliant blue R-250 (Sigma Aldrich). Extracted haemoglobin was stored at 4°C until use. Acid denatured haemoglobin was prepared by adjusting the pH of the extracted haemoglobin solution to pH 1.6 with 10.2 M hydrochloric acid and incubating at room temperature for 10 minutes prior to use in haemoglobinolysis assays.

Haemoglobinolysis assays were initially carried out over a pH range of 3.5–8.5. Sodium citrate was used for making the buffer of pH 3.5, sodium acetate for buffers of pH 4.5 and 5.5, sodium phosphate for buffers of pH 6.5 and 7.5 and HEPES for the buffer of pH 8.5. Reactions contained 100 ug acid denatured haemoglobin, 19 µg whole scabies mite extract and 100 µM of the relevant buffer in a final volume of 60 µL. Reactions were incubated at 37°C for 5 minutes prior to the addition of the substrate then incubated at 37°C for 2 hours. Protein bands were visualised by running on a 15% non-reducing SDS-PAGE and staining with coomassie brilliant blue R-250.

### Sequence identification and analysis

An EST dataset of over 40,000 human scabies mite (*S. scabiei* var. *hominis*) sequences [26], [27] was analysed using BLASTx [28] against the GenBank non-redundant database. A single contig of 10 sequences was shown to have homology to known aspartic proteases. As the contig lacked the 5‵ end of the gene sequence, the sequence was completed using a PCR and sequencing based approach on cDNA libraries using a combination of contig specific and vector primers. The complete coding sequence was submitted to GenBank (accession number KC540783). Signal sequence prediction was made using SignalP-HMM [29]. The amino acid sequence was compared to available sequences on GenBank using BLASTp [28].

### Preparation of enzymatically active recombinant *S. scabiei* aspartic protease

The sequence of the *S. scabiei* aspartic protease truncated by 23 amino acids at the N-terminus (designated N23SsAP) was amplified from a scabies mite cDNA library [26], [27] using the primers (5′-3′) F- *gCggATCC*AAATTATTACgAgTCAAATTgC and R- *gCCTgCAg*TCATTgTgATTgCgTAATg yielding a 1191 bp product. The primers included restriction enzyme sites (italicised in primer sequences), to facilitate directional cloning into pQE-9 in frame with the N-terminal hexa-histidine tag. The plasmid was transformed and expressed in *E.coli* strain BL21/pREP4. Cell pellets were resuspended in 50 mM Tris, 150 mM NaCl, 1 mM MgCl2, at a concentration of 1 g cells/5 ml buffer. The cells were lysed by sonication and inclusion bodies were isolated by layering 18 ml of the cell lysate over 10 ml of 27% sucrose solution followed by centrifugation for 45 minutes at 12000×g. The pellet was then resuspended in 50 mM Tris, 150 mM NaCl, 1 mM MgCl2, 1% Triton X-100 and passed again through a 27% sucrose solution as described above. The inclusion bodies were resuspended in 20 mM Tris buffer pH 8.0 at a concentration of 100 mg/ml. Inclusion bodies were solubilised at 2 mg/ml for 2 hours in denaturation buffer (8M urea, 50 mM Tris, 1 mM DTT, 150 mM NaCl, 10 mM imidazole, pH 8.5), then the pH adjusted to 7.0 with 4M HCl. The denatured protein solution was then centrifuged at 8000×g for 10 minutes to sediment any precipitates.

A Ni-NTA (Qiagen, Chadstone, Victoria, Australia) column was equilibrated with five column volumes of the solubilisation buffer. 25 mg solubilised inclusion bodies were loaded on the column and mixed for one hour at room temperature. The column was washed with 10 ml of 8M urea, 50 mM Tris, 1 mM DTT, 150 mM NaCl, 20 mM imidazole, pH 8.5. Bound histidine-tagged protein was eluted in 1.5 ml fractions with 8M urea, 20 mM Tris, 1 mM DTT, 150 mM NaCl, 250 mM imidazole, pH 8.5. The purified protein was concentrated using an stirred cell concentrator with 10 kDa molecular weight cut-off (MWCO) filter discs (Millipore, Billerica, MA, USA) to a final protein concentration of 2 mg/ml. The denatured protein was refolded by adding the protein drop wise to cold refolding buffer (20 mM NaH_2_PO4, 20 mM NaCl, 0.3 mM oxidised glutathione, 3 mM reduced glutathione, 300 mM L-arginine, pH 6.4) over a period of 2 hours to a final protein concentration of 20 µg/ml, with the refolding buffer stirring at a slow speed at 4°C. The refolding mix was left standing at 4°C for 24 hours, then the protein was concentrated in a 10 kDa MWCO centrifuge filter device. The concentration of the protein was determined by Bradford assay.

The refolded protein was activated by incubating the zymogen in 0.1M sodium citrate buffer pH 3.5and the activated protease was visualised by silver staining after separation by SDS-PAGE. A total of 11 different pH conditions were tested for the determination of the optimal pH for enzymatic activity of the activated N23SsAP using the fluorogenic substrate assay described above. Sodium citrate was used for buffers of pH 2.5–5.5, sodium phosphate for buffers of pH 6–7.5 and Tris for buffers of pH 8–9. The highest initial enzyme velocity was designated as 100% and enzyme velocities at other pHs were calculated as a percentage of this maximum value.

### Digestion of human haemoglobin and serum albumin by recombinant N23SsAP

Each 200 µl reaction contained 225 µg human haemoglobin or 62.5 µg human serum albumin, 2.5 µg refolded N23SsAP and 0.1M sodium citrate buffer pH 3.5. The recombinant enzyme was activated by incubating with the buffer for five minutes at 37°C prior to the addition of substrate. The reactions were then incubated at 37°C and samples taken at regular intervals and analysed on a 15% non-reducing SDS-PAGE gel.

### Digestion of human skin macromolecules by recombinant N23SsAP

Each 80 µl reaction contained 0.1M sodium citrate buffer pH 3.5, 0.3 µg refolded N23SsAP, 5 µM pepstatin A (where used) and 10 µg of one of the following substrates: fibrinogen; fibronectin; collagen III; or laminin. All the substrates were of human origin (Sigma Aldrich). In the reactions where enzyme activity was required to be blocked, pepstatin A was added to the activated enzyme reaction for five minutes before the addition of the substrate. The reactions were incubated at 37°C and samples were removed after 4 hours and 13 hours and analysed on 12% or 15% non-reducing SDS-PAGE gels.

## Results

### Analysis of aspartic protease activity in whole scabies mite extract

**Figure 1:**
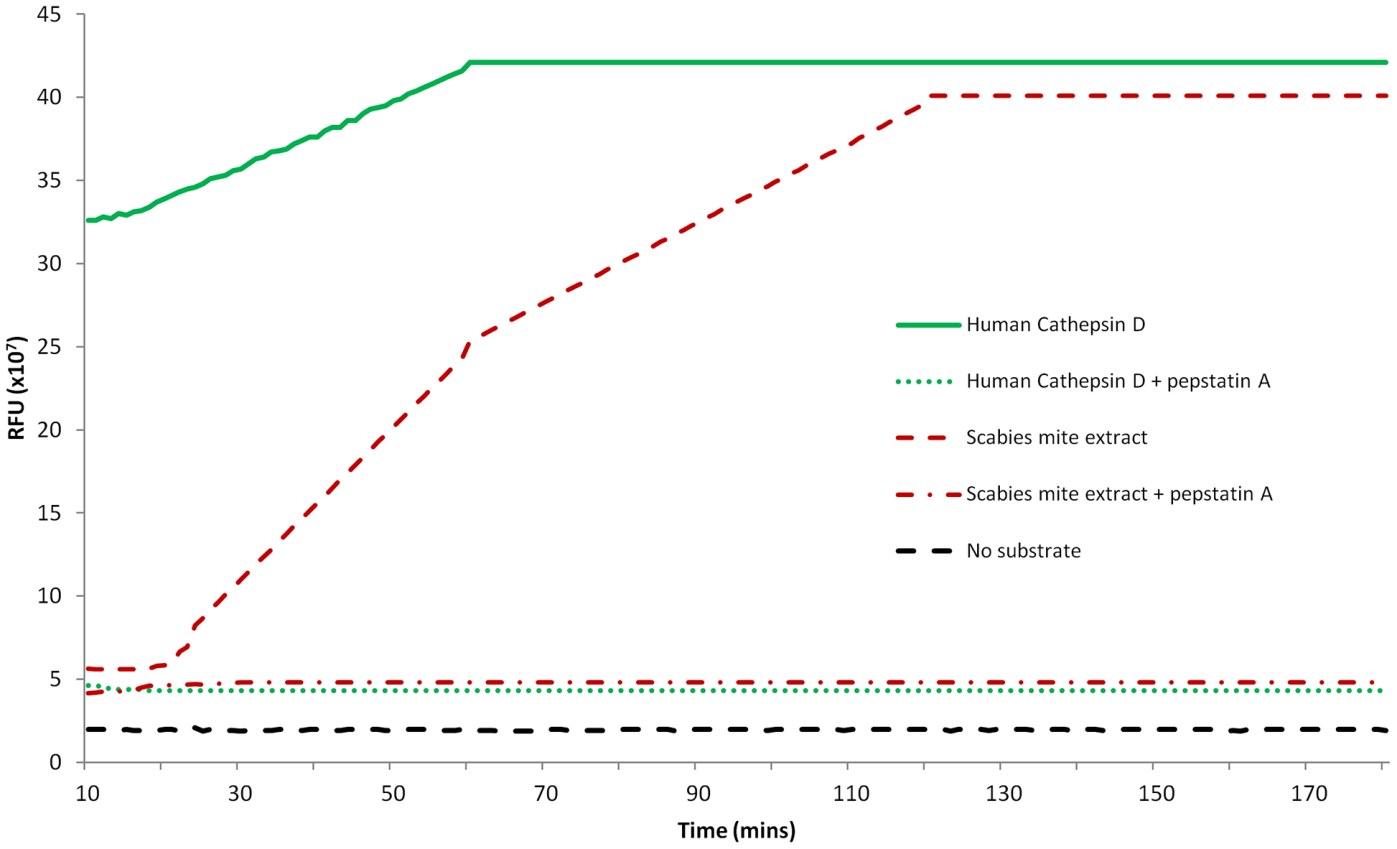
Detection of aspartic protease activity in scabies mite extract using a fluorescent substrate. The fluorescence signal was recorded at 60 second intervals, commencing after 10 minutes.

Whole scabies mite extract was initially examined for evidence of aspartic protease activity using a general aspartic protease fluorogenic substrate. Reactions were conducted at pH 3.5 as most aspartic proteases are active at an acidic pH. The human aspartic protease cathepsin D showed cleavage of the fluorescent substrate immediately, reaching maximum cleavage after 60 minutes, with cleavage completely blocked by the general aspartic protease inhibitor pepstatin A (Figure 1). Cleavage of the fluorescent substrate by the scabies mite extract was first detected after 18 minutes and reached the level of cleavage of human cathepsin D after two hours. Cleavage of the fluorogenic substrate by the scabies mite extract was blocked by pepstatin A, indicating the presence of aspartic protease activity in the whole mite extract.

**Figure 2:**
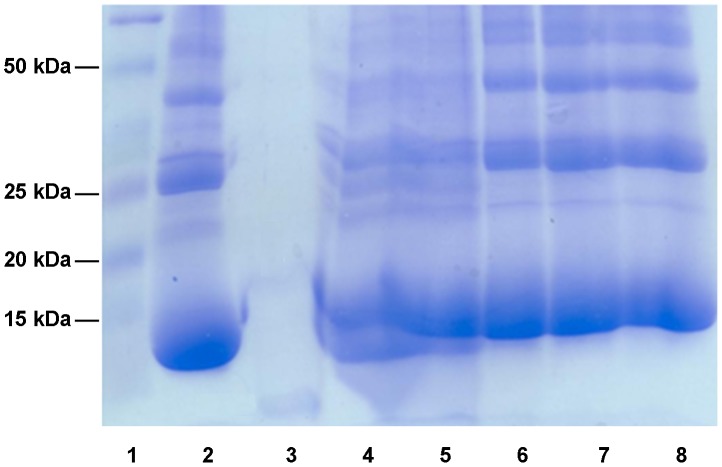
Digestion of acid denatured haemoglobin by scabies mite extract at pH–8.5. Haemoglobinolytic activity in whole human scabies mite extract was detected using acid denatured human haemoglobin (AD-Hb) as substrate at pH 3.5–8.5. Reactions were run on a 15% non-reducing SDS-PAGE gel and stained with coomassie brilliant blue R-250. Lane 1, molecular weight marker; lane 2, AD-Hb; lanes 3–8, AD-Hb plus scabies mite extract at pH 3.5, 4.5, 5.5, 6.5, 7.5 and 8.5 respectively.

The ability of scabies mite extract to digest human haemoglobin was then investigated. Reactions carried out with acid denatured haemoglobin over a pH range of 3.5–8.5 showed that almost complete digestion of acid denatured haemoglobin occurred at pH 3.5, with little or no digestion observed at pH 4.5 or above (Figure 2). Thus digestion of haemoglobin by the scabies mite extract occurred almost exclusively at an acidic pH consistent with digestion by aspartic and or cysteine proteases, as has been shown for related organisms such as ticks [14].

### *S. scabiei* aspartic protease sequence identification and analysis

To identify gene(s) encoding aspartic protease activity in the scabies mite, we analysed a *S. scabiei* var. *hominis* expressed sequence tag dataset [26], [27]. A single contig with homology to aspartic proteases from other organisms was identified. The sequence was predicted to encode a prepro-enzyme of 419 amino acids with a mass of 46.28 kDa, and a signal peptide of 22 amino acids. The deduced amino acid sequence shows many of the distinguishing features of the A1 family of aspartic proteases including two homologous lobes each containing a conserved DTG motif and the conservation of six cysteine residues predicted to form three characteristic disulphide bonds [30]. Comparison of the scabies mite aspartic protease pro-enzyme sequence with sequences available in GenBank showed that it had greatest sequence identity to cathepsin D aspartic proteases including from other Acari, such as the Blo t allergen of the storage mite *Blomia tropicalis* (AAX33731, 61% amino acid identity) and the cathepsin D of the tick *Ixodes scapularis* (XP_002416518, 54% amino acid identity). Based on the cleavage site of the pro-segment of human cathepsin D, the scabies mite aspartic protease has a pro-segment that is 52 amino acids in length yielding a 345 amino acid mature polypeptide.

### Preparation of enzymatically active N23SsAP

**Figure 3:**
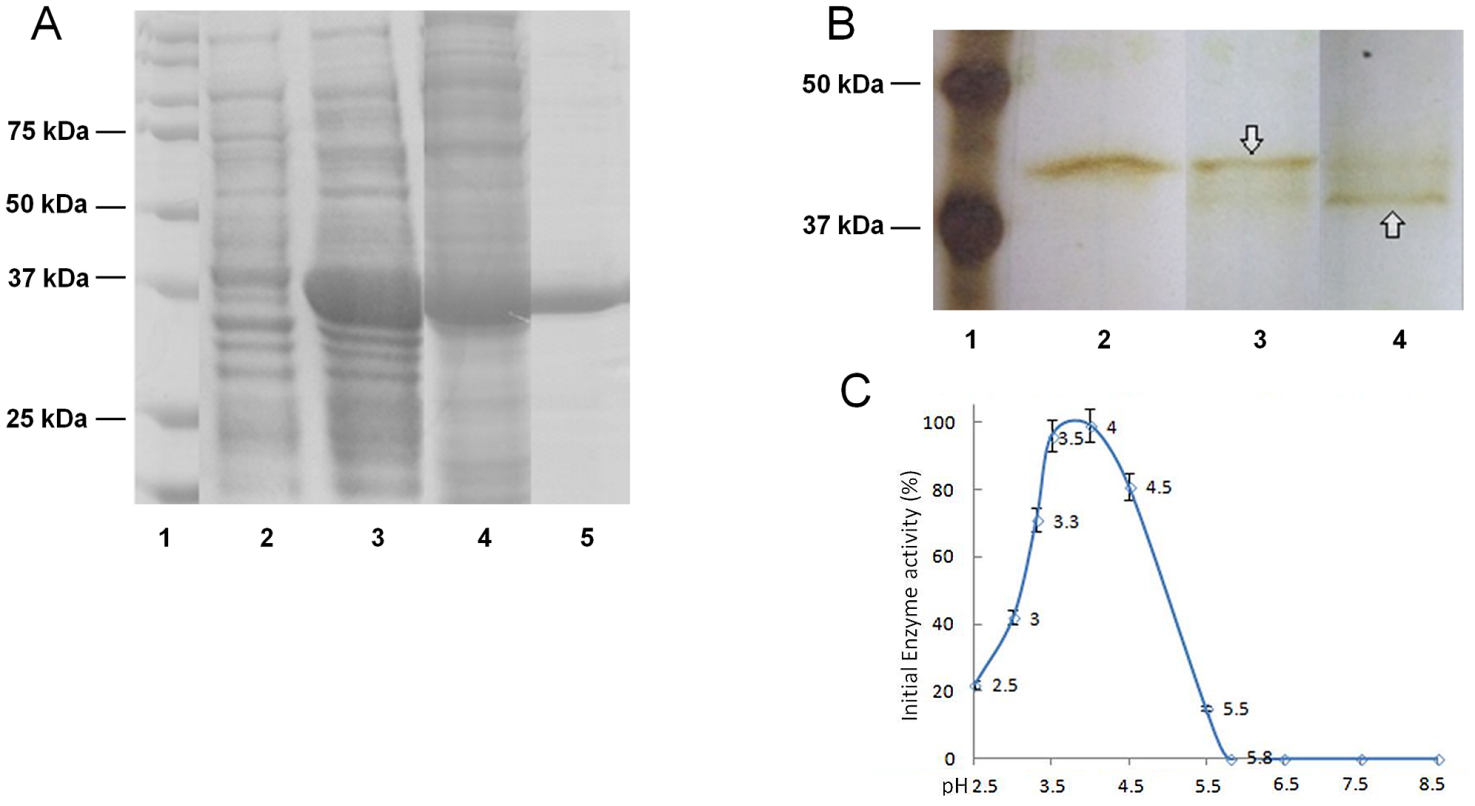
Production of enzymatically active recombinant N23SsAP. A. N23SsAP was expressed in *E. coli* and purified over Ni-NTA. The expressed and purified protein was analysed by SDS-PAGE stained with coomassie brilliant blue R-250. Lane 1, molecular weight marker; lane 2, *E. coli* lysate before induction of the protein expression; lane 3, *E. coli* lysate after induction of the protein expression; lane 4, flow through from the Ni-NTA column; lane 5, purified N23SsAP eluted from the Ni-NTA column. B. N23SsAP (lane 2) was incubated at pH 3.5 and samples taken after 15 (lane 3) and 30 minutes (lane 4). Activation of the zymogen was visualised by silver staining after SDS-PAGE. Downward pointing arrow- zymogen; upward pointing arrow- activated enzyme. C. The activity of the activated enzyme at different pH conditions was measured using a fluorogenic substrate. The highest initial enzyme velocity was designated as 100% and enzyme velocities at other pHs were calculated as a percentage of this maximum value.

Recombinant *S. scabiei* aspartic protease lacking the first 23 amino acids of the full protein sequence (N23SsAP) was produced in *E. coli*. The protein was expressed in inclusion bodies and purified over Ni-NTA (Figure 3A). N23SsAP was refolded using a rapid dilution method and self-activated at pH 3.5 (Figure 3B). The activity of the recombinant N23SsAP was determined using the fluorescent substrate assay. N23SsAP shows the characteristic pH profile of an aspartic protease, with maximum activity in the range of pH 3.5–4.5 (Figure 3C).

### Digestion of blood and skin macromolecules by recombinant *S. scabiei* aspartic protease

**Figure 4:**
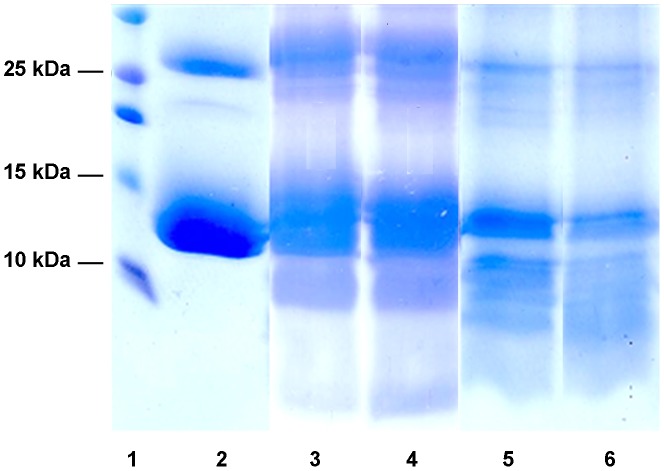
Digestion of human haemoglobin by the recombinant human scabies mite aspartic protease N23SsAP. The haemoglobinolytic activity of N23SsAP was detected using native human haemoglobin as substrate at pH% non-reducing SDS-PAGE gel and stained with coomassie brilliant blue R-250. Lane 1, molecular weight marker; lane 2, haemoglobin alone; lanes 3–6, haemoglobin plus N23SsAP, incubated for 1 hour (lane 3), 2 hours (lane 4), 4 hours (lane 5) or 12 hours (lane 6).

**Figure 5:**
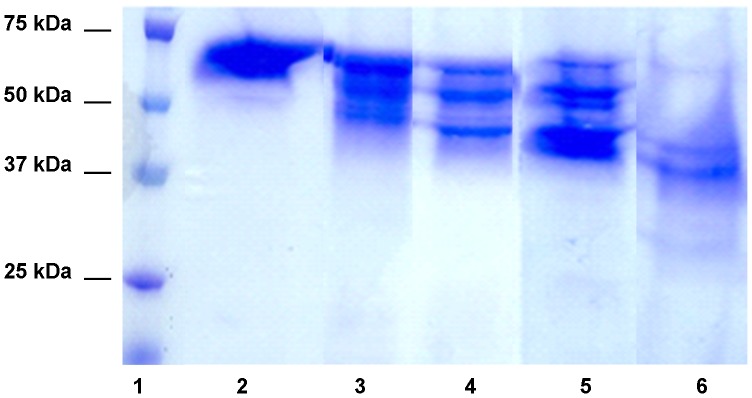
Digestion of human serum albumin by the recombinant human scabies mite aspartic protease N23SsAP. Recombinant N23SsAP was tested for its ability to digest human serum albumin. Reactions were run on a 15% non-reducing SDS-PAGE gel and stained with coomassie brilliant blue R-250. Lane 1, molecular weight marker; lanes 2–6, serum albumin plus N23SsAP, incubated for 15 minutes (lane 2), 1 hour (lane 3), 2 hours (lane 4), 4 hours (lane 5) or 12 hours (lane 6).

**Figure 6:**
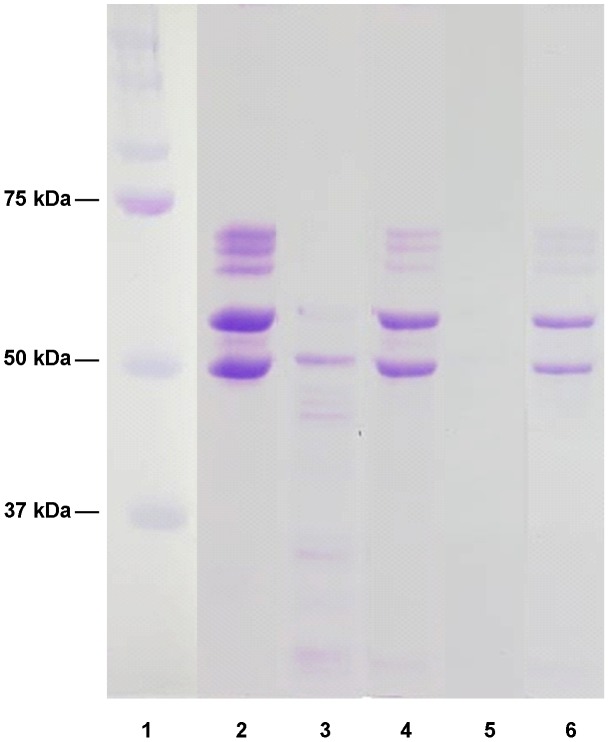
Digestion of human fibrinogen by the recombinant human scabies mite aspartic protease N23SsAP. Recombinant N23SsAP was tested for its ability to digest human fibrinogen. Reactions were run on a 15% non-reducing SDS-PAGE gel and stained with coomassie brilliant blue R-250. Lane 1, molecular weight marker; lane 2, fibrinogen alone; lanes 3 and 5, fibrinogen plus N23SsAP; lanes 4 and 6, fibrinogen plus N23SsAP and pepstatin A, after 4 hours incubation (lanes 3 and 4) and 13 hours incubation (lanes 5 and 6).

**Figure 7:**
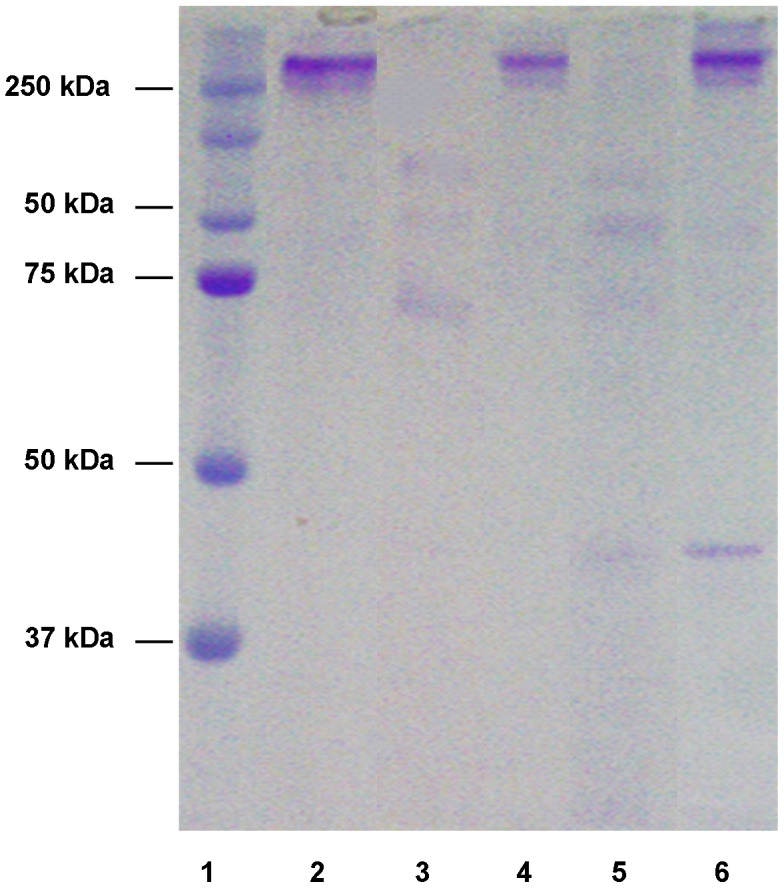
Digestion of human fibronectin by the recombinant human scabies mite aspartic protease N23SsAP. Recombinant N23SsAP was tested for its ability to digest human fibronectin. Reactions were run on a 15% non-reducing SDS-PAGE gel and stained with coomassie brilliant blue R-250. Lane 1, molecular weight marker; lane 2, fibrinonectin alone; lanes 3 and 5, fibrinonectin plus N23SsAP; lanes 4 and 6, fibrinonectin plus N23SsAP and pepstatin A, after 4 hours incubation (lanes 3 and 4) and 13 hours incubation (lanes 5 and 6).

**Figure 8:**
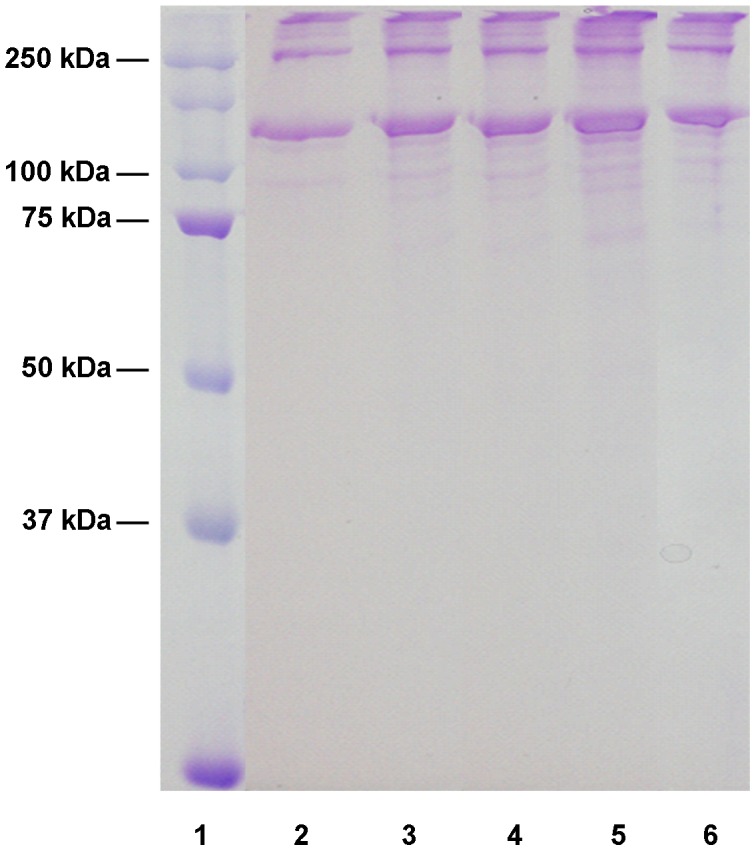
Digestion of human collagen III by the recombinant human scabies mite aspartic protease N23SsAP. Recombinant N23SsAP was tested for its ability to digest human collagen III. Reactions were run on a 15% non-reducing SDS-PAGE gel and stained with coomassie brilliant blue R-250. Lane 1, molecular weight marker; lane 2, collagen III alone; lanes 3 and 5, collagen III plus N23SsAP; lanes 4 and 6, collagen III plus N23SsAP and pepstatin A, after 4 hours incubation (lanes 3 and 4) and 13 hours incubation (lanes 5 and 6).

A number of physiologically relevant blood and skin macromolecules were then tested as substrates for N23SsAP. Digestion of native human haemoglobin into smaller peptides was apparent after one hour of incubation with N23SsAP, with further digestion apparent after 12 hours (Figure 4). Evidence of digestion human serum albumin by N23SsAP was also apparent after one hour, with considerable digestion observed after 12 hours (Figure 5). Undigested fibrinogen appeared as a series of bands between 75 and 50 kDa on a 15% SDS-PAGE gel (Figure 6, lane 2). After four hours of incubation with N23SsAP, much of the fibrinogen was digested to a size less than 50 kDa (lane 3). After 13 hours incubation, fibrinogen had undergone substantial digestion (lane 5). The fibrinogen digestion by N23SsAP was inhibited by pepstatin A (lanes 4 and 6). Similarly, the large molecular weight fibronectin showed significant digestion by N23SsAP after 4 and 13 hours. This digestion was largely inhibited by the addition of pepstatin A (Figure 7). No digestion of collagen III (Figure 8) or laminin (data not shown) was observed, with the substrates remaining intact after 13 hours incubation with N23SsAP.

## Discussion

This study details the first analysis of aspartic protease activity in scabies mites. The presence of aspartic protease activity in scabies mite extract was clearly shown using both a fluorescent peptide and human haemoglobin as substrates. The lag in the activity of the scabies mite extract supports the possibility that the scabies mite aspartic protease being present as a zymogen in the mite extract (Figure 1). The observed lag might also be due to an unmatched specificity of the scabies mite aspartic protease to the fluorogenic substrate. The higher rate of cleavage by human cathepsin D might be due to the fact that the cleavage of the fluorescent substrate is between a Phe-Phe bond, which matches the specificity of human cathepsin D. While the A1 family of aspartic proteases generally has a cleavage site between two hydrophobic residues, neither the concentration nor the specificity of the aspartic protease present in the scabies mite extract is currently known. The preference of scabies mite extract to digest haemoglobin at an acidic pH is consistent with aspartic protease activity.

A single *S. scabiei* aspartic protease gene was identified, with sequence analysis revealing that the gene encoded a cathepsin D like enzyme. Despite subsequent analysis of a large EST dataset and PCR based approaches using degenerate primers designed to the highly conserved active site regions on scabies mite cDNA and DNA, no further genes encoding aspartic proteases were identified (data not shown). Thus while the presence of further genes encoding aspartic proteases cannot be ruled out, the present data suggest that scabies mites may possess a single aspartic protease gene. This is in contrast to other mammalian parasites such as ticks, hookworms, schistosomes and malaria parasites, where aspartic proteases are expressed as a family of enzymes. For example, although there is high sequence identity among the plasmepsin family of aspartic proteases of the malaria parasite *Plasmodium*, their substrate specificity and response to inhibitors differ [31]. Of the three aspartic protease paralogues identified in the tick *Ixodes ricinus*, only one is exclusively expressed in the gut, where it is involved in haemoglobin digestion [32], indicating other potential roles for the other paralogues. Thus competitive and selective inhibition of aspartic protease activity in these parasites is a challenging task. The presence of a single aspartic protease in the scabies mite would not present such complexity.

In this study, recombinant *S. scabiei* aspartic protease N23SsAP was shown to digest human haemoglobin, serum albumin, fibrinogen and fibronectin. A previous study of enzymatic activity in scabies mite extract, which did not investigate aspartic protease activity, reported the absence of lipase activity in the scabies mite extract pointing towards a non-lipid source of nutrition [8]. Although the exact source of nutrition for scabies mites has not been conclusively demonstrated, abundant proteins such as haemoglobin and serum albumin are expected to be major sources of amino acids. Canine scabies mites have been shown to have longer *in vitro* survival times on diets that contain serum [33]. Similar to *Psoroptes* mites, the digestion of fibronectin and fibrinogen by scabies mites could perform an anti-coagulation function ensuring the availability of serous material containing albumin and haemoglobin. The recombinant N23SsAP was unable to digest human collagen III and laminin in these experiments. These skin molecules form part of the basement membrane and may represent the boundary that restricts scabies mites to the epidermal layer of human skin.

The role of aspartic proteases in pathogenic processes has been established for a number of parasites. The essential nature of these enzymes in the lifecycle of some parasites has made them targets of drug discovery and vaccine development efforts. For example, monoclonal antibodies against the *R. microplus* yolk pro-cathepsin inoculated into cattle were able to produce partial protection against challenge with *R. microplus* larvae [34]. Subsequent vaccination trials with recombinant yolk pro-cathepsin induced an overall protection of 25% against challenge with *R. microplus* larvae [35]. An aspartic protease from the human hookworm *N. americanus* was shown to be efficacious as a vaccine against subsequent hookworm challenge in dogs with lower adult parasite burdens and reduced host blood loss observed in vaccinated animals [36], [37]. Peptides from the *N. americanus* aspartic protease induced neutralising antibodies in mice that inhibited the enzymatic activity of the protease [38], [39]. The development of novel therapeutics for scabies is of increasing importance given the evidence of emerging resistance to current treatments. We have shown that a scabies mite aspartic protease is able to digest human skin and serum molecules, raising the possibility that interference with the function of the enzyme may impact on mite survival.
